# 

**Published:** 1890-09-27

**Authors:** 


### NOTICE TO CORRESPONDENTS.

All MS., Letters, Books for Review, and other matters intended for the Editor should be addressed The Editor, The Lodge, Porchester Square, London, W.

The Editor cannot undertake to return rejected M.S., even when accompanied by stamped directed envelope.

### Notice to Advertisers and Subscribers.

All Advertisements, Orders for Copies of The Hospital, and Business Communications should be addressed to The Publisher, *not to the Editor*, The Hospital, 140, Strand, W.C.

### Bound Volumes of "The Hospital."

Vol. VI., containing full page portraits of T.R.H. the Prince and Princess of Wales, and Miss Florence Nightingale, is obtainable at 4s., post free.

Orders will be received for Vol. VII., 4s. post free. Cases for binding may be had of the Publisher, at 1s. 6d. each.

### To Residents, Secretaries, and Matrons.

The Editor will be glad to have the Regulations and each Report as issued by Asylums, Hospitals, Convalescent and Nursing Institutions, as well as those of other Charities, and an early copy in duplicate of all Appeals and Festival Notices, etc., addressed to *The Editor, The Lodge, Porchester Square, London, W.* Any superintendent, secretary, matron, or other chief official who regularly sends such information may be registered, on application to the Publisher, to receive free of cost a cover for binding The Hospital in half-yearly volumes.

### BURDETT'S HOSPITAL ANNUAL FOR 1890

**Is now ready, and may be obtained of the Publisher, The Hospital Offices, 140, Strand, W.C., price 2s. 6d. limp boards, or 3s. 6d. cloth. All orders must be accompanied by a remittance.**

### Students' Column.

### UNIVERSITY OF DURHAM.

September, 1890.

SECOND EXAMINATION FOR THE DEGREE OF BACHELOR IN MEDICINE.

Honours—*Second Class:* Hoyle, J. C., M.R.C.S. Eng., L.R.C.P. Lond., St. Bartholomew's Hospital; Marson, F. H., Queen's College, Birmingham; Hollick, J. O., Queen's College, Birmingham; May, B., College of Medicine, Newcastle-upon-Tyne; Peacock, W. E., College of Medicine, Newcastle-upon-Tyne; Peacock, E. V., College of Medicine, Newcastle-upon-Tyne.

The following candidates have satisfied the Examiners: Best, H. G., Middlesex Hospital; Bond, B. W., St. Thomas's Hospital; Browne, F. D., Yorkshire College, Leeds; Caddy, D. J., College of Medicine, Newcastle-upon-Tyne; Case, H., College of Medicine, Newcastle-upon-Tyne; Cass, E. E., Yorkshire College, Leeds; Claridge, H. A. H., Queen's College, Birmingham; Dawson, H. K., College of Medicine, Newcastle-upon-Tyne; De Silva, Appu-Hennedige C.. Ceylon Medical College; Dow, W. A., St. Bartholomew's Hospital; Gardner, A. J., M.R.C.S. Eng., L.R.C.P. Ed., Yorkshire College, Leeds; Green, R., College of Medicine, Newcastle-upon-Tyne; Greenyer, V. T., St. Bartholomew's Hospital; Hawthorn, F., College of Medicine, Newcastle-upon-Tyne; Hodgins, W. W., University College, London; Hodson, T. G. S., London Hospital; Hunter, J. H., College of Medicine, Newcastle-upon-Tyne; Jones, F. S., London Hospital; Keenan, T. F., College of Medicine, Newcastle-upon-Tyne; McNabb, A. A. J., College of Medicine, Newcastle-upon-Tyne; Meaden, C., Bristol Medical School; Mitchell, E., College of Medicine, Newcastle-upon-Tyne; Shepard, R. H., St. Bartholomew's Hospital; Simpson, F. H., Queen's College, Birmingham: Spicer, H., St. Bartholomew's Hospital; Stephenson, J. B., Middlesex Hospital; Sterling, R., B.A., L.Th., College of Medicine, Newcastle-upon-Tyne; Titterton, H. C., Queen's College, Birmingham; Walch, C. N. C., St Bartholomew's Hospital; Whillis, S. S., College of Medicine, Newcastle-upon-Tyne; Williams, G. J., College of Medicine, Newcastle-upon-Tyne.

EXAMINATION FOR THE LICENCE IN SANITARY SCIENCE.

September, 1899.

The following candidates have satisfied the examiners: Barras, W. G., M.B., Glasgow, D.P.H., Glasgow; Griffith, W. B., L.R.C.S., L.R.C.P., Ed.; Hutton, J. A., M.B., B.S., Durh., M.R.C.S., Eng., L.R.C.P., Lond.; Richardson, J. N., M.D., Durh., M.R.C.S.; Robinson, J., M.D., Brussels, M.R.C.S., Eng., L.R.C.P., Ed.

### GENERAL CHARITIES.

[This Column is devoted to an account of work of charitable institutions other than Medical Charities. The Editor will be glad to receive reports or news of work at 140, Strand. *Philanthropy* should be plainly written on the envelope.]

---

THE Educational Committee of the **LONDON YOUNG WOMEN'S CHRISTIAN ASSOCIATION** have issued a very excellent prospectus for their winter evening classes. Book-keeping, shorthand, type-writing, cookery, ambulance, dressmaking, are taught, and pupils are prepared for the Civil Service. At about twenty of the institutes in London, prizes and certificates are given to those successful in examination. Physical exercises are not forgotten, and gymnastic drill is held at several of the institutes. Any further information may be had from the Secretary, 16A, Old Cavendish Street, W.

Thanks partly to the kindness of the "Australians" rendering their services gratuitously for the occasion of the benefit match, the sum of nearly £700 will be added to the **CRICKETERS' FUND.**

People who have to live on small incomes prefer to pay up weekly for their necessaries rather than trust to the dubious possibility of saving enough for the quarterly bill, for somehow the money goes before the bill arrives. For this reason the Birmingham Gas Department mean to try an experiment of giving gas to artizans' dwellings by the automatic penny-in-the-slot principle. Three-pence a 100 feet is now the charge for small consumers, and by the new apparatus it will be twenty-five feet for a penny, which charge, however, will include fittings and meter. This pre-payment meter acts so that if the householder likes to put 9d. into the slot he can have 225 cubit feet supply, according to the money put in, the valve rises, and as the supply decreases the valve closes. It is certainly very enterprising of Birmingham, and anything which might lessen the dangerous use of paraffin lamps would be a boon. Then against that, there is the ill effect of gas in small rooms. Anyway we are glad to see any effort which will bring daily comfort to the smaller classes of dwellings.

Though a good many of the general public have never heard of **THE MENDICITY SOCIETY** the magistrates of the Metropolitan Police Courts know well what valuable work is done by them. To take the opinion of one, Sir John Bridge writes: "I much desire to bear testimony to the valuable work done by the society in clearing the streets of beggars and preventing poor children being dragged out in bad weather in order to assist in wringing contributions from those who are both benevolent and foolish." This society has been going ever since 1818. It aims at discouraging money relief to street beggars, it rescues the unfortunate children employed by professional beggars, and investigates begging letters in town and country. Whenever a man is sent to prison convicted through the instrumentality of the society, it will find him work on his discharge, thus giving him a chance of becoming an honest man if he is willing. The children who are rescued are sent to industrial schools. Subscribers to this society are supplied with food tickets with which they can give relief to cases of distress met with in the streets. If money were never given to street-beggars their number would decrease with extraordinary rapidity, since money wherewith to get drink and live a life of idleness is the aim and object of the professional beggar who sometimes makes from five to six shillings a day. There is simply no end to the blandishment and devices of the modern mendicant, but there are people still who believe any got-up story, who would decline to give to some authenticated good work, but who open their purse to a shabby-looking man with his arm tied up who assures them that "he had the misfortune to fall from a scaffold." Those whose hearts are of this touching description can scarcely do better than call at the Mendicity Society's office, where they can hear many surprising facts about the professional mendicant. This society affords prompt and effectual assistance to those whom sudden calamity and unaffected distress may cast in want and misery, and many a man and woman have been helped along by this timely assistance. Where there are such numbers of charities so direfully in need of support it is very exasperating to reflect on the pounds and pounds thrown away daily on undeserving wretches, but the public is soft-hearted and lazy, and never reflects on the evil that odd pennies can do. There is a museum at the office of the society where the stock in trade of beggars of many years may be seen. Sketches of the different accidents which the impostor alleges have disabled him, or sketches of terrific operations by the surgeon's knife which he has had to undergo (with an admiring group of medical students looking on), are some of the means by which the beggar works upon his victims' feelings. Catalogues of woe on a placard are common, everyday sights. This is a good authentic specimen. "Please friend to assist a poor dumb man who had his tongue cut out by four Spaniards, and also subject to fits. Good hearing."! The last income of the society was £1,445. We recommend charitable folk who want to help the deserving and not the undeserving to consult the Mendicity society, whose great object is *prompt assistance*. There is such a thing as taking so long to investigate the genuineness of a poor man's appeal, that he dies meanwhile from starvation. Secretary, Eric Buchanan, Esq., 8, Fisher Street, Red Lion Square, Holborn.

